# Oral Dexmedetomidine Achieves Superior Effects in Mitigating Emergence Agitation and Demonstrates Comparable Sedative Effects to Oral Midazolam for Pediatric Premedication: A Systematic Review and Meta-Analysis of Randomized Controlled Studies

**DOI:** 10.3390/jcm13041174

**Published:** 2024-02-19

**Authors:** Chun-Kai Jen, Kuo-Ching Lu, Kuan-Wen Chen, Yun-Ru Lu, I-Tao Huang, Yu-Chen Huang, Chun-Jen Huang

**Affiliations:** 1 Department of Anesthesiology, Wan Fang Hospital, Taipei Medical University, 111, Sec. 3, Xinglong Rd., Wenshan Dist., Taipei 116, Taiwan; 109228@w.tmu.edu.tw (C.-K.J.); 64536@w.tmu.edu.tw (K.-C.L.); 109227@w.tmu.edu.tw (K.-W.C.); 109229@w.tmu.edu.tw (Y.-R.L.); 2 Integrative Research Center for Critical Care, Wan Fang Hospital, Taipei Medical University, Taipei 116, Taiwan; 3 Department of Anesthesiology, Binkun Women’s and Children’s Hospital, Taoyuan 324, Taiwan; 4 Emergency Department, Redcliffe Hospital, Redcliffe, QLD 4020, Australia; itao.huang@uq.net.au; 5 School of Public Health, Faculty of Medicine, University of Queensland, Herston, QLD 4006, Australia; 6 Department of Dermatology, Wan Fang Hospital, Taipei Medical University, Taipei 116, Taiwan; 99185@w.tmu.edu.tw; 7 Research Center of Big Data and Meta-Analysis, Wan Fang Hospital, Taipei Medical University, Taipei 116, Taiwan; 8 Department of Dermatology, School of Medicine, College of Medicine, Taipei Medical University, Taipei 116, Taiwan; 9 Graduate Institute of Clinical Medicine, College of Medicine, Taipei Medical University, Taipei 116, Taiwan; 10 Department of Anesthesiology, School of Medicine, College of Medicine, Taipei Medical University, Taipei 116, Taiwan

**Keywords:** dexmedetomidine, midazolam, premedication, pediatric, emergence agitation, sedation

## Abstract

**Background**: Oral midazolam is the most commonly used sedative premedication agent in pediatric patients. While effective, oral midazolam cannot reduce the incidence of emergence agitation. Oral dexmedetomidine may be effective in providing satisfactory sedation and reduce the incidence of emergence agitation, although the results of different randomized controlled trials are conflicting. **Methods**: This study enrolled randomized controlled trials (RCTs) examining premedication with oral dexmedetomidine versus oral midazolam in pediatric patients undergoing general anesthesia. PubMed, the Cochrane Library, Embase, and the Web of Science database were searched from their inception until June 2023. The outcomes were the incidence of satisfactory preoperative sedation, satisfactory sedation during separation from parents, satisfactory sedation during anesthesia induction using an anesthesia mask, and the incidence of emergence agitation. **Results**: A total of 9 RCTs comprising 885 patients were analyzed. Our data revealed comparable effects of dexmedetomidine and midazolam with respect to satisfactory preoperative sedation and a satisfactory incidence of sedation during parental separation and mask acceptance before anesthesia induction. Notably, our data revealed that the rate of emergence agitation was significantly lower in pediatric patients receiving dexmedetomidine (*n* = 162) than in those receiving midazolam (*n* = 159) (odds ratio = 0.16; 95% confidence interval: 0.06 to 0.44; *p* < 0.001; *I*^2^ = 35%). **Conclusions**: Data from this meta-analysis revealed comparable effects for premedication with oral dexmedetomidine or oral midazolam with respect to satisfactory sedation; furthermore, premedication with oral dexmedetomidine more effectively mitigated emergence agitation in pediatric patients receiving general anesthesia compared with oral midazolam.

## 1. Introduction

Perioperative anxiety is common in pediatric patients, and untreated anxiety can lead to difficult induction of anesthesia, increased postoperative pain, and even emergence agitation [1]. Sedative premedication is therefore often used to increase children’s cooperation for a smooth induction of anesthesia [2,3]. Oral midazolam is among the most commonly used sedative premedication agents in pediatric patients and is regarded by many as the gold standard for premedication [2]. Midazolam is a short-acting gamma-aminobutyric acid receptor agonist with sedative, anxiolytic, and anterograde amnesic effects [4]. Although this drug is safe and effective, preoperative administration of oral midazolam does not reduce the incidence of postoperative emergence agitation [5,6]. One proposed explanation for this finding is that oral midazolam has a duration of action of less than 2 h, and hence the effect of oral midazolam given preoperatively may be diminished by the end of surgery [7].

Dexmedetomidine is a highly selective and potent alpha-2 adrenoreceptor agonist with both sedative and analgesic effects [8,9]. Dexmedetomidine has a more gradual onset of action and a longer duration of action than midazolam [10,11]. Premedication with both intranasal and intravenous dexmedetomidine can provide satisfactory preoperative sedation in pediatric patients [12]. Moreover, premedication with both intranasal and intravenous dexmedetomidine has been found to reduce the incidence of emergence agitation in pediatric patients [6,12]. While effective, both intranasal and intravenous premedication may be difficult to administer in uncooperative children; children are more accepting of the less invasive and less irritative oral route of administration [2]. Although the effectiveness of oral dexmedetomidine may be in question because of its high first-pass effect and poor oral bioavailability [10], several randomized controlled trials (RCTs) have demonstrated that premedication with oral dexmedetomidine provides satisfactory sedation not inferior to that of oral midazolam [13,14,15,16,17]. A limitation of these RCTs is that of small sample size [13,14,15,16,17]. 

Emergence agitation is frequently observed in pediatric patients during early recovery from general anesthesia. Emergence agitation involves behaviors such as restlessness, disorientation, excitation, non-purposeful movement, inconsolability, and thrashing [1,6]. The incidence of emergence agitation varies from 20% to 80%, with factors such as age, assessment tool, definition, anesthetic technique and type of surgery [1,6]. Though previous studies revealed that several pharmacological and non-pharmacological approaches can exert certain beneficial effects on preventing emergence agitation in pediatric patients receiving general anesthesia, the optimal combinations are still lacking to date [1,6]. Of note, pre-emptive treatment with sedative or anxiolytic agents has been proposed. However, as mentioned earlier, oral midazolam, the gold standard of premedication, fails to demonstrate a consistent effect on reducing emergence agitation [1,5,6]. Oral dexmedetomidine, with its analgesic and sympatholytic effects, may theoretically reduce the incidence of emergence agitation [10,18]. Unfortunately, several RCTs comparing the effectiveness of oral dexmedetomidine versus oral midazolam for preventing emergence agitation have yielded inconclusive results, possibly attributable to the relatively small sample sizes of the RCTs included [14,16,19,20,21].

This systematic review and meta-analysis compared the effectiveness of premedication with oral dexmedetomidine versus premedication with oral midazolam. The two primary aims of this study were to examine the effectiveness of oral dexmedetomidine in providing satisfactory preoperative sedation when compared to oral midazolam, and to determine whether oral dexmedetomidine can effectively reduce the incidence of emergence agitation when compared to oral midazolam.

## 2. Materials and Methods

### 2.1. Protocol

We employed a systematic approach to identify publications that evaluated the efficacy and safety of oral dexmedetomidine premedication for pediatric patients. This systematic review and meta-analysis were implemented in accordance with the Preferred Reporting Items for Systematic Reviews and Meta-Analyses 2020 and the Cochrane review methods [22]. The protocol of this systematic review was registered in the International Prospective Register of Systematic Reviews (CRD42023389445, registration date: 5 January 2023).

### 2.2. Data and Literature Sources

The authors of this study searched Embase, PubMed, the Cochrane Central Register of Controlled Trials, and the Cochrane Database of Systematic Reviews from their inception until 30 June 2023. We also implemented a literature search of the Web of Science and Google Scholar to identify relevant studies. Only articles published in English were evaluated. Search was done using the following keywords: (“children” OR “child” OR “pediatrics” OR “pediatric”) AND (“dexmedetomidine”) AND (“oral form” OR “premedication” OR “oral administration”). Only human RCTs were included. In addition, the reference lists of relevant studies and review articles were also manually searched to identify additional studies.

### 2.3. Study Selection

Two reviewers (C.K.J. and K.C.L.) independently evaluated the identified studies using predefined selection criteria. A third reviewer (Y.C.H. or C.J.H.) adjudicated if any disagreements arose between the two reviewers during the primary study selection. The following studies fulfilling the following criteria were enrolled in this meta-analysis: (1) RCTs published in international journals and written in English; (2) studies whose participants were children (patients under 18 years old) undergoing premedication treatment before surgery; (3) studies whose intervention was premedication with oral dexmedetomidine versus oral midazolam; and (4) studies whose outcomes examined the sedative effects of the premedication, such as the incidence of satisfactory sedation before induction, the incidence of satisfactory sedation during parent separation, the incidence of satisfactory sedation during mask induction of anesthesia, and the incidence of emergence agitation. Any adverse effects (e.g., nausea and vomiting, nasal irritation, laryngospasm, and/or shivering, etc.) were also recorded.

### 2.4. Data Extraction

Two reviewers (C.K.J. and K.C.L.) independently extracted data using a prespecified data extraction form. A third reviewer (Y.C.H. or C.J.H.) then verified the extracted data. The following variables were extracted: (1) the number of patients and patient characteristics; (2) the protocol for premedication administration, such as timing, agent, and dose; (3) the diagnostic criteria for emergence agitation and the definitions for satisfactory sedation, parental separation, and mask induction; (4) the incidence of outcomes; and (5) the incidence of adverse events and hemodynamic effects. If these variables were not reported in an article, we emailed the authors to request the relevant data.

### 2.5. Assessment of Methodological Quality

Two reviewers (C.K.J. and K.C.L.) independently assessed the risk of bias using the Cochrane risk-of-bias tool, which considered the methods of random sequence generation, allocation concealment, blinding of participants and the outcome estimator, incomplete reporting of outcome data, selective reporting of outcomes, and other potential sources of bias. The quality of each RCT was independently rated by two authors (C.K.J. and K.C.L.). A senior researcher (Y.C.H. or C.J.H) adjudicated if disagreements arose between the two authors (C.K.J. and K.C.L.) relating to the quality assessments of articles, until a consensus was reached.

### 2.6. Statistical Analysis

Binary outcomes were reported using odds ratios (ORs) with 95% confidence intervals (CIs). Heterogeneity between studies was assessed using the *I*^2^ statistic [23]. An *I*^2^ statistic of >50% was indicative of substantial data heterogeneity among the studies. We employed random effects models when significant statistical or clinical heterogeneity was detected. In addition, we implemented subgroup analysis to determine the potential factors contributing to significant heterogeneity and to reduce its effect. Studies with an unclear or high risk of bias in more than one area were excluded from the analysis. All statistical analyses were implemented using the Cochrane Collaboration’s Review Manager software (RevMan version 5.4). We also used the Grading of Recommendations, Assessment, Development, and Evaluation (GRADE) method to assess the overall quality of evidence. This approach incorporates factors such as study limitations, inconsistency of effects, imprecision, indirectness, and publication bias [24].

## 3. Results

### 3.1. Study Selection

**Figure 1:**
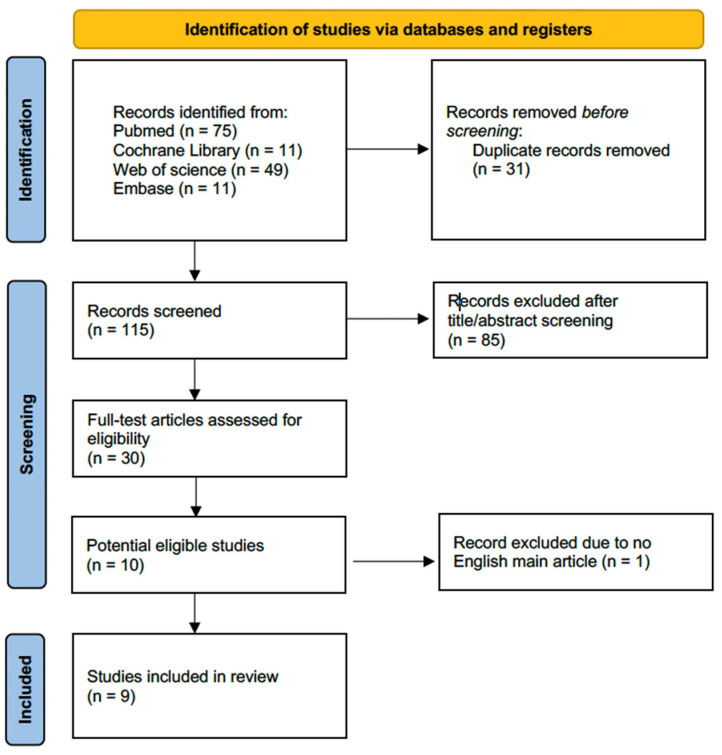
Flowchart of the database search and selection process based on Preferred Reporting Items for Systematic Reviews and Meta-Analyses guidelines.

A total of 146 publications were identified in the initial database search. We removed 31 duplicate articles and further excluded 85 articles by screening their titles and abstracts. After reviewing the full manuscripts of the remaining 30 publications, we identified 10 potentially relevant studies. One remaining study was eliminated because the main article was written in Spanish. Consequently, this meta-analysis enrolled 9 RCTs involving a total of 885 individuals (Figure 1).

### 3.2. Study Characteristics and Patient Populations

The characteristics of the enrolled studies are summarized in Table 1. The studies were undertaken between 2011 and 2022 in four countries: the United States (one), Turkey (one), Iran (one), and India (six) [13,14,15,16,17,19,20,21,25]. All studies compared the effectiveness of oral dexmedetomidine with that of oral midazolam. All studies were RCTs involving pediatric patients who underwent various procedures under general anesthesia (e.g., dental restoration procedure, urogenital procedure, esophageal dilation, minor lower abdominal surgery, ophthalmic surgery, and congenital heart surgery). The patients’ ages ranged from 1 to 12 years, and most were aged from 1 to 7 years. All patients were classified as having American Society of Anesthesiologists physical status I or II, with the exception of those in one study [25]. Premedication was administered 30–60 min before the patients were brought to the operating room for anesthesia induction. All the patients in the midazolam group received an oral midazolam dose of 0.5 mg/kg, whereas the patients in the dexmedetomidine group received either 2, 2.5, or 4 μg/kg oral dexmedetomidine. One study compared two doses of oral dexmedetomidine (i.e., 2 or 4 μg/kg) [17]. Two studies also included oral clonidine [13,15], and one study compared oral dexmedetomidine with oral melatonin and midazolam [20]. All patients received volatile anesthetics for the maintenance of anesthesia.

### 3.3. Quality of the Included Studies

**Figure 2:**
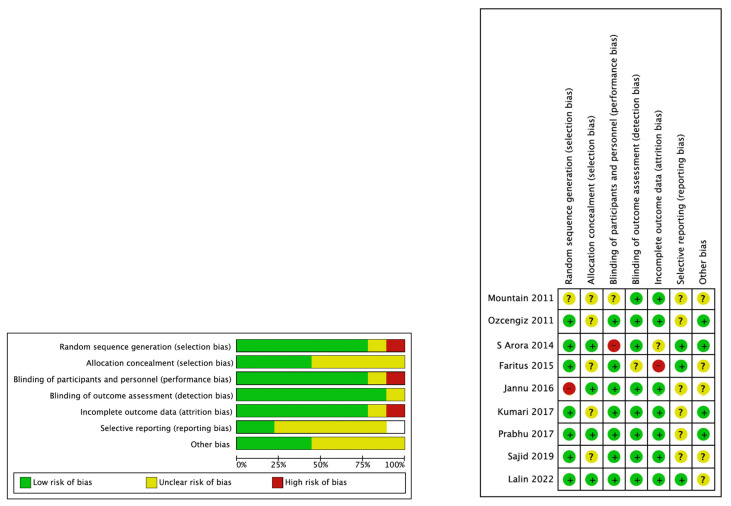
Summary of risk of bias: review authors’ judgements about each risk of bias item for each included study. Green indicates a low risk of bias, yellow indicates some concerns of bias, and red indicates a high risk of bias [13,14,15,16,17,19,20,21,25].

All studies used a random allocation method. Most studies had a low risk of incomplete and selective reporting of outcome data. Risk of bias graphs and summaries are presented in Figure 2. 

### 3.4. Meta-Analysis Results

#### 3.4.1. Incidence of Satisfactory Sedation

**Figure 3:**
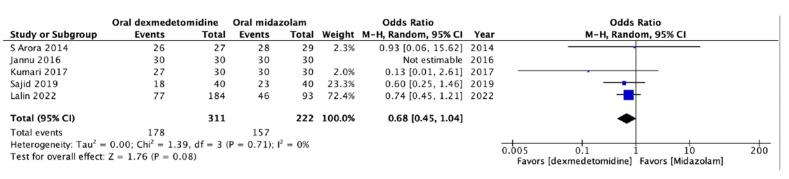
Forest plot of the meta-analysis of the incidence of satisfactory sedation using oral dexmedetomidine or oral midazolam as premedication. The square shown for each study (first author and year of publication) is the odds ratio (OR) for individual trials, and the corresponding horizontal line is the 95% confidence interval (CI). The lozenge-shaped symbol at the bottom indicates pooled OR with 95% CI [13,14,15,16,17].

A satisfactory sedation outcome was reported in five RCTs with 533 patients [13,14,15,16,17,25]. The level of sedation was evaluated using either a 3- [15], 4- [13,14,17], or 5-point [16] scale, and a predefined scoring system was used to classify patients into satisfactory or unsatisfactory sedation groups in each study. Our results revealed that the rate of satisfactory preoperative sedation did not differ significantly between the patients receiving oral dexmedetomidine or oral midazolam for premedication (OR = 0.68; 95% CI: 0.45 to 1.04; *p* = 0.08; *I*^2^ = 0%; Figure 3). The data indicated the comparable effectiveness of oral dexmedetomidine and oral midazolam with respect to satisfactory preoperative sedation.

#### 3.4.2. Incidence of Satisfactory Sedation during Parental Separation

**Figure 4:**
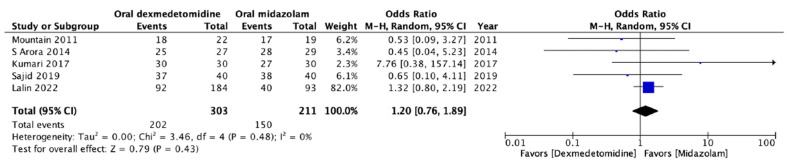
Forest plot of the meta-analysis of the incidence of satisfactory parental separation using oral dexmedetomidine or oral midazolam as premedication. The square shown for each study (first author and year of publication) is the odds ratio (OR) for individual trials, and the corresponding horizontal line is the 95% confidence interval (CI). The lozenge-shaped symbol at the bottom represents the pooled OR with 95% CI [13,15,16,17,21].

Satisfactory sedation during parental separation was reported in five RCTs involving 514 patients [13,15,16,17,21]. All analyzed studies used the Parental Separation Anxiety Scale (PSAS) to evaluate parental separation, and a PSAS score less than 2 was regarded as acceptable separation from parents. Our results revealed that the rate of satisfactory parental separation did not differ significantly between the patients who received oral dexmedetomidine or oral midazolam for premedication (OR: 1.20; 95% CI: 0.76 to 1.89; *p* = 0.43; *I*^2^ = 0%; Figure 4). The data indicated the comparable effectiveness of oral dexmedetomidine and oral midazolam with respect to satisfactory sedation during parental separation.

#### 3.4.3. Incidence of Satisfactory Sedation during Mask Induction

**Figure 5:**
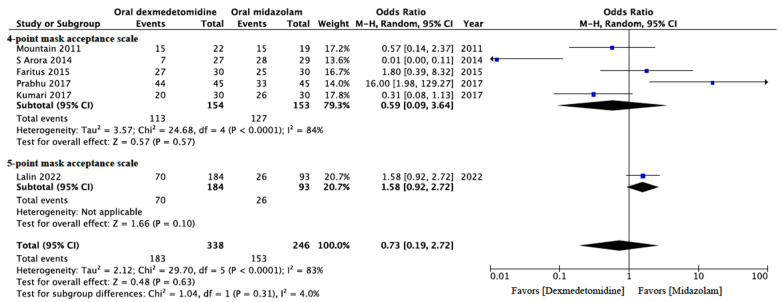
Forest plot of the meta-analysis of the incidence of satisfactory mask acceptance during induction with premedication using oral dexmedetomidine or oral midazolam. The top forest plot is for the subgroup analysis of studies using the 4-point mask acceptance score (MAS). The lowest lozenge-shaped symbol is incidence of satisfactory mask acceptance during induction with premedication using oral dexmedetomidine or oral midazolam for all studies. The square shown for each study (first author and year of publication) is the odds ratio (OR) for individual trials, and the corresponding horizontal line is the 95% confidence interval (CI). The lozenge-shaped symbol indicates the pooled OR with 95% CI [13,15,17,19,21,25].

Six RCTs comprising 584 patients assessed satisfactory sedation during mask induction [13,15,17,19,21,25]. Sedation status during mask induction was evaluated using a 4- [13,15,19,21,25] or 5-point [17] mask acceptance scale. Sedation acceptance was regarded as either excellent (i.e., unafraid, cooperative, and accepting mask easily) or good (i.e., cooperative with slight resistance but easily reassured) during mask induction. The data indicated that the rate of satisfactory sedation during mask induction did not differ significantly between the participants who received oral dexmedetomidine and those who received oral midazolam for premedication (OR: 0.73; 95% CI: 0.19 to 2.72; *p* = 0.63; *I*^2^ = 83%; Figure 5). The data also revealed that oral dexmedetomidine and oral midazolam yielded comparable effects with respect to achieving satisfactory sedation for mask induction. However, an *I*^2^ value of 83% indicated significant data heterogeneity among the studies. We implemented a subgroup analysis of the studies that used a 4-point mask acceptance scale [13,15,19,21,25] to reduce heterogeneity; the results of the subgroup analysis were similar to the initial results, but significant heterogeneity remained (OR: 0.59; 95% CI: 0.09 to 3.64; *p* = 0.57; *I*^2^ = 84%; Figure 5).

#### 3.4.4. Incidence of Emergence Agitation

**Figure 6:**
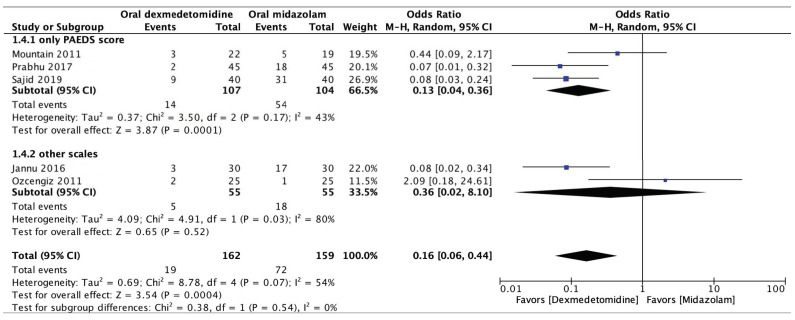
Forest plot of the meta-analysis of the incidence of emergence agitation with oral dexmedetomidine or oral midazolam. The top forest plot is from a subgroup analysis of studies using the pediatric anesthesia emergence delirium scale (PAEDS). The lowest lozenge-shaped symbol is the incidence of emergence agitation with the use of oral dexmedetomidine or oral midazolam for all studies. The square shown for each study (first author and year of publication) is the odds ratio (OR) for individual trials, and the corresponding horizontal line is the 95% confidence interval (CI). The lozenge-shaped symbol represents the pooled OR with 95% CI [14,16,19,20,21].

The incidence of emergence agitation was extracted from five RCTs comprising 321 patients [14,16,19,20,21]. Emergence agitation was evaluated using postoperative agitation scores [14], the emergence agitation scale (EAS) [20], or the pediatric anesthesia emergence delirium scale (PAEDS) [16,19,21]. The results revealed a significantly lower rate of emergence agitation in patients receiving oral dexmedetomidine than in those receiving oral midazolam for premedication (OR: 0.16; 95% CI: 0.06 to 0.44; *p* < 0.001; *I*^2^ = 54%, Figure 6), indicating that premedication with oral dexmedetomidine more effectively mitigated emergence agitation than premedication with oral midazolam. The results indicated significant data heterogeneity among the studies, with an *I*^2^ of 54%. To reduce heterogeneity, we implemented a subgroup analysis of studies that used the PAEDS to assess emergence agitation [16,19,21]. The results of the subgroup analysis agreed with the initial results but had lower heterogeneity (OR: 0.13; 95% CI: 0.04 to 0.36; *p* < 0.001; *I*^2^ = 43%; Figure 6).

### 3.5. Adverse Effects

Mean pulse oximetry values or the incidence rates of hypoxemia were reported in four studies [14,17,20,21]. The results indicated no difference in mean pulse oximetry value between the patients receiving oral dexmedetomidine and those receiving oral midazolam, and no hypoxemia was reported. With respect to haemodynamic effects, Kumari and colleagues observed lower mean blood pressure in patients receiving oral dexmedetomidine than in those receiving oral midazolam, when measured at 45–60 min after administration (65.72 versus 71.28 mm Hg; *p* < 0.001) [15]. Prabhu and colleagues had similar findings [19]. Notably, no patients in the dexmedetomidine group required medical treatment. Other studies have revealed no statistically significant differences in mean blood pressure or heart rate between patients receiving oral dexmedetomidine and oral midazolam [14,17,20,21]. None of the analyzed RCTs reported significant hypotension or bradycardia that required treatment in either group during the study period.

### 3.6. GRADE Findings

GRADE assessments are summarized in Table 2. The following outcomes were evaluated: the incidence of emergence agitation, satisfactory sedation, satisfactory sedation during parental separation, and satisfactory sedation during mask acceptance. Two outcomes had a high level of certainty, namely the incidence of satisfactory sedation and the incidence of satisfactory sedation during parental separation. Two outcomes had a moderate level of certainty, namely the incidence of satisfactory sedation during mask acceptance and the incidence of emergence agitation.

## 4. Discussion

Midazolam is considered the gold standard for premedication in pediatric patients because of its safety and ease of oral administration [3,26]. Data from the present systematic review and meta-analysis revealed that premedication with oral dexmedetomidine and oral mediation can achieve comparable outcomes for pediatric patients undergoing general anesthesia with respect to satisfactory sedation, parental separation, and mask acceptance. Moreover, the results revealed that premedication with oral dexmedetomidine can more effectively mitigate emergence agitation in pediatric patients undergoing general anesthesia when compared to premedication with oral midazolam. These results have meaningful clinical implications, as the sample sizes of the included outcome measurements are large (range: 321 to 584 pediatric patients), and meta-analyses of RCTs are at the top of evidence level hierarchies.

The oral form of dexmedetomidine is highly acceptable to pediatric patients as premedication because it is colorless, odorless, and tasteless [16]. However, compared with midazolam, dexmedetomidine has a slower onset of action. The mean onset time of oral dexmedetomidine ranges from 23 (5 μg/kg) to 42 (3 μg/kg) minutes and varies by dose [27]. Therefore, premedication with oral dexmedetomidine must be administered earlier than midazolam to achieve satisfactory sedation before induction of anesthesia. Although oral dexmedetomidine has a slower onset than oral midazolam, data from the analyzed RCTs [14,15,16,17,18,20,21,22,26] indicated that 2–4 μg/kg of oral dexmedetomidine can provide satisfactory preoperative sedation within 45 min. Current data therefore indicates that oral dexmedetomidine should be administered approximately 45 min before anesthesia induction to allow sufficient time for the medication to take effect and provide satisfactory sedation in pediatric patients during induction.

Because of its sympatholytic effects, dexmedetomidine may cause bradycardia and hypotension, posing potential safety concerns [12]. In a study comparing premedication with oral dexmedetomidine to oral ketamine, oral dexmedetomidine with a dose of 3–5 μg/kg was associated with a dose-dependent reduction in heart rate and systolic blood pressure, with a maximum decrease of up to 20% compared to baseline values [27]. However, this reduction in heart rate and blood pressure was within a tolerable range, and no patients in that study required clinical intervention [27]. Moreover, all patients in that study had stable pulse oximetry values [27]. The results of that study are consistent with those of the RCTs [14,15,17,19,20,21] in this meta-analysis. The present data support the assertion that oral dexmedetomidine with a dose ranging from 2–4 μg/kg can provide reliable preoperative sedation without significant side effects. Of note, ketamine is proposed to be effective in reducing emergence agitation with additional merits such as maintaining spontaneous breathing during sedation, reducing postoperative pain, and providing bronchodilation [1,6,27,28]. However, in a systemic review and meta-analysis conducted by Rao et al., ketamine is associated with a higher risk of emergence agitation compared to dexmedetomidine [29]. Clinicians should take this into consideration when considering administrating ketamine in pediatric patients.

Emergence agitation is among the most common complications in pediatric patients who undergo anesthesia [18]. The underlying cause of emergence agitation in pediatric patients remains undetermined, but possible contributing factors include pain, preoperative anxiety, increased sympathetic tone, and the use of short-acting volatile agents [18]. Preoperative administration of midazolam yielded inconsistent results for reducing the incidence of emergence agitation [12]. A meta-analysis of 37 studies of pediatric patients undergoing general anesthesia with volatile anesthetic agents also indicated that midazolam has a non-significant role in preventing emergence agitation [29]. This finding may be related to its relatively short duration of action (i.e., less than 2 h) [30]. Another possible explanation is its lack of analgesic effect [30]. This concept is supported by a study revealing that effective treatment of postoperative pain reduced the incidence of emergence agitation [18]. Dexmedetomidine, on the other hand, possesses sympatholytic and analgesic effects [2]. Moreover, the duration of action of dexmedetomidine is longer than that of midazolam (i.e., an elimination half-life of 2–3 h) [31]. These characteristics may all contribute to the prevention of emergence agitation; therefore, oral dexmedetomidine can be reasonably assumed to be more effective in reducing emergence agitation when compared to midazolam.

This meta-analysis has several limitations. First, significant heterogeneity among the studies was found. The factors contributing to this heterogeneity may include the dose of medication and the time of its administration, the type of surgery, and variations in age range. Furthermore, various sedation scales and measurements impeded further data analysis. Second, only five–six RCTS were included in the outcome analyses of sedation. Similarly, only five RCTs were included in the outcome analysis of emergence agitation, all of which were conducted in a single country (India). Additional RCTs and data from multiple countries are required to validate the applicability of these results in the general population. Moreover, we did not perform a trial sequential analysis [32,33] during conduction of our protocol. Therefore, the question of whether the observed effects are large enough and will not be affected by further studies remains to be elucidated. Third, the dose and time of administration of oral dexmedetomidine varied in the included studies. Because the optimal dose of oral dexmedetomidine remains undetermined, the development of an effective protocol that ensures both the desired efficacy and the avoidance of possible side effects is ongoing. Fourth, some clinically relevant outcomes such as the amnesic effect of oral dexmedetomidine, and parental or patients’ satisfaction of oral dexmedetomidine compared to oral midazolam, are not investigated in current available literature according to our research. Future studies focusing on the above topics may be needed to further shed light on the benefits of oral dexmedetomidine use. Finally, although most of the studies had a low risk of bias, some studies had an unclear or high risk of bias. Therefore, these findings must be interpreted with caution.

## 5. Conclusions

This systematic review and meta-analysis revealed that oral dexmedetomidine is an effective premedication agent. Premedication with oral dexmedetomidine can achieve superior effects in mitigating emergence agitation and demonstrates comparable sedative effects to oral midazolam for pediatric patients undergoing general anesthesia. Oral dexmedetomidine has no clinically significant side effects when compared to oral midazolam, and a dose of 2–4 μg/kg is likely a safe and reliable option for premedication in children.

## Figures and Tables

**Table 1:** Baseline characteristics of randomized controlled trials.

Study *	Country	Jadad Score	*n*	Dexmedetom-idine (Dose)	Midazolam (Dose)	Outcomes	Age (Years)	Type of Surgery	ASA	Anesthesia	Timing of Premedication
Mountain (2011) [21]	United States	5	41	4 μg/kg	0.5 mg/kg	Parental separation anxiety scale (PSAS) ^a^ Mask acceptance scale (MAS) ^b^ Pediatric anesthesia emergence delirium scale (PAEDS) ^c^	1–6	Dental restoration procedure	I, II	Sevoflurane, O_2_, and N_2_O	30 min
Ozcengiz (2011) [20]	Turkey	5	100	2.5 μg/kg	0.5 mg/kg	Hemodynamic variables Emergence agitation scale (EAS) ^d^	3–9	Oesophagealdilatation procedure	I, II	Sevoflurane 8%, N_2_O 50%, and O_2_	40–45 min
Arora (2014) [13]	India	5	85	4 μg/kg	0.5 mg/kg	4-point sedation scale ^e^ Parental separation anxiety scale (PSAS) ^a^ Mask acceptance scale (MAS) ^b^	1–4	Elective urogenital procedure	I, II	Sevoflurane followed by isoflurane	30 min for midazolam 60 min for dexmedetomi-dine
Faritus (2015) [25]	Iran	4	60	2 μg/kg	0.5 mg/kg	Ramsay sedation scale (not included because of unclear data analysis) Mask acceptance scale (MAS) ^b^	2–12	Congenital heart surgery	III	Sevoflurane	45 min
Jannu (2016) [14]	India	5	60	4 μg/kg	0.75 mg/kg	4-point sedation scale ^f^ Parental separation anxiety scale (not included due to insufficient data) 5-point mask acceptance scale ^g^ (not included due to insufficient data) Emergence agitation ^h^	1–7	Elective minor lowerabdominal surgery	I, II	Sevoflurane, O_2_, and N_2_O	40 min
Kumari (2017) [15]	India	5	90	4 μg/kg	0.5 mg/kg	3-point sedation scale ^i^ Parental separation anxiety scale (PSAS) ^a^ Mask acceptance scale (MAS) ^b^	4–12	Elective ophthalmic surgery	I,II	Halothane	30 min
Prabhu (2017) [19]	India	5	90	4 μg/kg	0.5 mg/kg	Mask acceptance scale (MAS) ^b^ Emergence agitation (PAEDS) ^a^	1–10	Elective surgery	I,II	Sevoflurane	45 min
Sajid (2019) [16]	India	5	80	4 μg/kg	0.5 mg/kg	5-point sedation scale ^j^ Parental separation anxiety scale (PSAS) ^a^ Intravenous acceptability score Pediatric anesthesia emergence delirium scale (PAEDS) ^c^	1–6	Elective herniotomy	I	Isoflurane	40 min
Lalin (2022) [17]	India	5	279	2 or 4 μg/kg	0.5 mg/kg	4-point sedation scale ^f^ Parental separation anxiety scale (PSAS) ^a^ 5-point mask acceptance scale ^g^ Postoperative agitation score (not included due to insufficient data)	1–7	Pyeloplasty, hernia surgery, urethral surgery, ureteral reimplant, hypospadias	I,II	Sevoflurane +/− caudal block	45 min

* The studies are presented in order of year of publication. ^a^ Parental separation anxiety scale (PSAS): Grade 1 = easy separation; Grade 2 = whimpers but is easily reassured and does not cling; Grade 3 = cries and cannot be easily reassured but does not cling; Grade 4 = cries and clings to parents; Grades 1 and 2 were considered to denote acceptable separation. ^b^ Mask acceptance scale (MAS): 1 = excellent (unafraid, cooperative, and accepts mask easily); 2 = good (slight fear of mask, easily reassured); 3 = fair (moderate fear of mask, not calmed with reassurance); 4 = poor (terrified, crying, or combative); scores of 1 and 2 were regarded as acceptable sedation. ^c^ Pediatric anesthesia emergence delirium scale (PAEDS). PAEDS comprises five criteria (i.e., the ability to make eye contact, purposeful action, awareness of surroundings, restlessness, inconsolability). Each criterion has a 5-point scale, and a score over 10 was considered emergence agitation. ^d^ Emergence agitation scale (EAS): 1 = awake, calm, and cooperative; 2 = crying and requires consoling; 3 = irritable, screaming, and inconsolable; 4 = combative, disoriented, thrashing; scores of 3 and 4 were considered to represent emergence agitation. ^e^ 4-point sedation scale: 1 = asleep; 2 = drowsy, responds to verbal commands or gentle stimulation; 3 = awake, calm, quiet; 4 = anxious, depressed, agitated, or crying; scores from 1 to 3 were considered to indicate satisfactory sedation. ^f^ 4-point sedation scale in this study: 1 = anxious, depressed, agitated, or crying; 2 = awake, calm, quiet; 3 = drowsy, responds to verbal commands or gentle stimulation; 4 = asleep; scores from 2 to 4 were considered to denote satisfactory sedation. ^g^ 5-point mask acceptance scale: 1 = combative, crying; 2 = moderate fear of mask, not easily calmed; 3 = cooperative with reassurance; 4 = calm, cooperative; 5 = asleep; scores from 3 to 5 were considered to denote satisfactory sedation. ^h^ Emergence agitation was assessed as 1 = agitated, crying; 2 = crying but easily consoled; and 3 = calm; a score of 1 was considered emergence agitation. ^i^ 3-point sedation scale: 1 = awake; 2 = drowsy; 3 = asleep; scores of 2 and 3 were considered to represent satisfactory sedation. ^j^ 5-point sedation scale: Grade 1: agitated; Grade 2: oriented, calm, and cooperative; Grade 3: drowsy but responsive to verbal commands; Grade 4: nonresponsive to verbal commands but responsive to painful stimuli; Grade 5: nonresponsive to painful stimuli; scores of 3 to 5 were considered to denote satisfactory sedation.

**Table 2:** Findings related to the Grading of Recommendations, Assessment, Development, and Evaluation approach.

Certainty Assessment						Number of Patients		Effect	Certainty
Number of Studies	Study Design	Risk of Bias	Inconsistency	Indirectness	Impression	Dexmedetomidine	Midazolam	Odds Ratio (95% CI)	
Incidence of satisfactory sedation									
5	RCT	not serious	not serious	not serious	not serious	311	222	0.68 (0.45–1.04)	High
Incidence of satisfactory sedation during parent separation									
5	RCT	not serious	not serious	not serious	not serious	303	211	1.20 (0.76–1.89)	High
Incidence of satisfactory sedation during mask acceptance									
6	RCT	not serious	serious ^a^	not serious	not serious	338	246	0.73 (0.19–2.72)	Moderate
Incidence of emergence agitation									
5	RCT	not serious	seriou ^a^	not serious	not serious	162	159	0.16 (0.06–0.44)	Moderate

^a^ Rated reduced because the *I*^2^ value was >50%.

## Data Availability

The data can be requested from the corresponding author.
